# Improving measurements of the falling trajectory and terminal velocity of wind‐dispersed seeds

**DOI:** 10.1002/ece3.9183

**Published:** 2022-08-04

**Authors:** Jinlei Zhu, Carsten M. Buchmann, Frank M. Schurr

**Affiliations:** ^1^ Institute of Landscape and Plant Ecology University of Hohenheim Stuttgart Germany

**Keywords:** diaspore, free fall with air resistance, inertia, mechanistic model, samara, seed dispersal by wind, seed falling velocity, terminal velocity

## Abstract

Seed dispersal by wind is one of the most important dispersal mechanisms in plants. The key seed trait affecting seed dispersal by wind is the effective terminal velocity (hereafter “terminal velocity”, *V*_*t*_), the maximum falling speed of a seed in still air. Accurate estimates of *V*_*t*_ are crucial for predicting intra‐ and interspecific variation in seed dispersal ability. However, existing methods produce biased estimates of *V*_*t*_ for slow‐ or fast‐falling seeds, fragile seeds, and seeds with complex falling trajectories. We present a new video‐based method that estimates the falling trajectory and *V*_*t*_ of wind‐dispersed seeds. The design involves a mirror that enables a camera to simultaneously record a falling seed from two perspectives. Automated image analysis then determines three‐dimensional seed trajectories at high temporal resolution. To these trajectories, we fit a physical model of free fall with air resistance to estimate *V*_*t*_. We validated this method by comparing the estimated *V*_*t*_ of spheres of different diameters and materials to theoretical expectations and by comparing the estimated *V*_*t*_ of seeds to measurements in a vertical wind tunnel. *V*_*t*_ estimates closely match theoretical expectations for spheres and vertical wind tunnel measurements for seeds. However, our *V*_*t*_ estimates for fast‐falling seeds are markedly higher than those in an existing trait database. This discrepancy seems to arise because previous estimates inadequately accounted for seed acceleration. The presented method yields accurate, efficient, and affordable estimates of the three‐dimensional falling trajectory and terminal velocity for a wide range of seed types. The method should thus advance the understanding and prediction of wind‐driven seed dispersal.

## Graphical abstract

Accurate estimates of seed terminal velocity, the key seed trait affecting seed dispersal by wind, are crucial for predicting intra‐ and interspecific variation in seed dispersal ability. However, existing methods produce biased estimates of terminal velocity for slow‐ or fast‐falling seeds, fragile seeds, and seeds with complex falling trajectories. We present a new video‐based method that yields accurate, efficient and affordable estimates of the three‐dimensional falling trajectory and terminal velocity for a wide range of seed types, and should thus advance the understanding and prediction of wind‐driven seed dispersal.

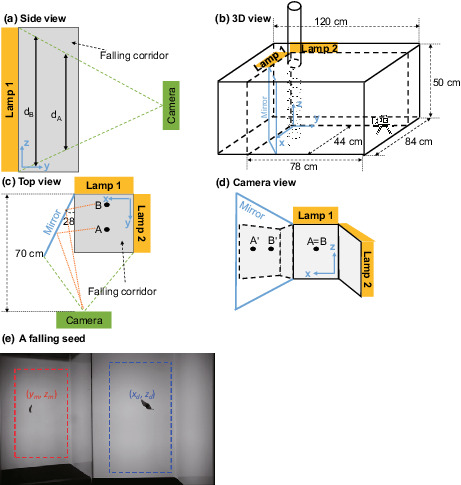

## 1 INTRODUCTION

Seed dispersal by wind is one of the most important and best understood dispersal mechanisms (Nathan et al., 2011). A seed (or diaspore) released from the mother plant starts falling to the ground and accelerates until it reaches a terminal falling velocity (*V*_*t*_) at which the opposing forces of gravitation and aerodynamic drag cancel out. *V*_*t*_ is the most important seed trait determining wind‐driven seed dispersal (Nathan et al., 2011). This is because seeds with lower *V*_*t*_ are transported further by horizontal wind components and are more likely to be uplifted by turbulent airflows that can disperse seeds over long distances (Higgins et al., 2003; Nathan et al., 2002, 2011). A method to measure *V*_*t*_ should ideally (1) accurately measure *V*_*t*_ of single seeds, (2) work for various seed types, (3) estimate *V*_*t*_ of seeds that still accelerate, and (4) be nondestructive so that seeds can be used for further measurements. To our knowledge, no existing method fulfills all these criteria.

Existing methods for measuring *V*_*t*_ fall into three categories. The first lets seeds accelerate over a certain distance and then estimates *V*_*t*_ as vertical seed velocity in a measuring section (Sullivan et al., 2018). This approach is nondestructive, but yields biased *V*_*t*_ estimates for seeds that still accelerate. A second method measures the air speed needed to suspend seeds in vertical air flow (Jongejans & Schippers, 1999). This avoids bias from seed acceleration. While the method can accurately measure the mean *V*_*t*_ of seed bulks, it is challenging to apply to single or slow‐falling seeds because it is difficult to accurately control and measure vertical wind speed (Russo, 2011). Vertical wind tunnels may also damage fragile seeds and are challenging to use for seeds with complex falling trajectories (such as auto‐rotating seeds). A third method includes video‐based approaches that work for different seed types (Loubet et al., 2007), and are nondestructive (Wyse et al., 2019).

**FIGURE 1:**
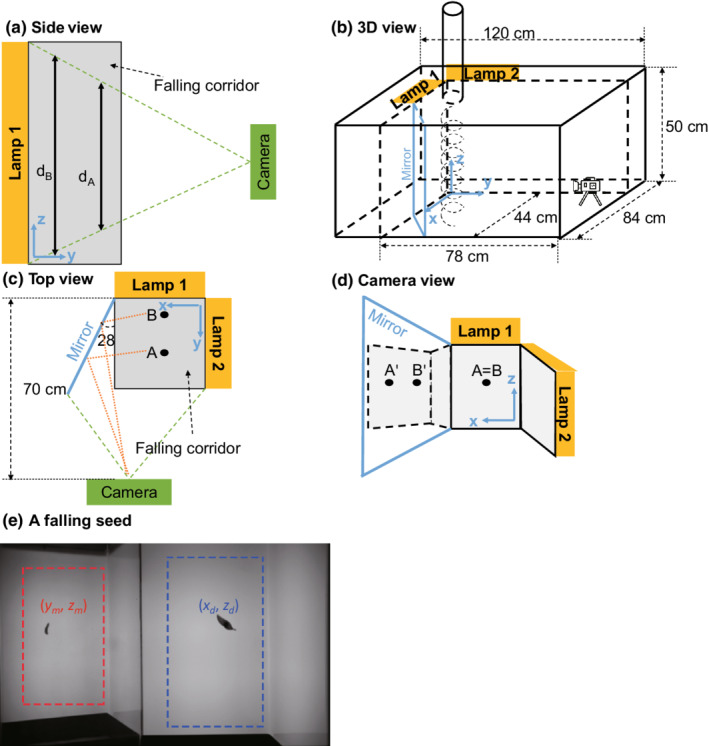
Video‐based measurement of three‐dimensional trajectories and terminal velocities (*V*_*t*_) of falling seeds. (a) Video‐based measurement faces the challenge that two seeds at different horizontal distances from the camera (A and B) may show the same pixel displacement between video frames even though they differ in true seed displacement (*d*_A_ and *d*_B_) and hence in falling velocity. (b) The presented apparatus circumvents this problem by using a mirror (light blue) that enables the camera to simultaneously record a seed from two perspectives. (c,d) Even though seeds A and B have identical positions in the direct image, their positions in the mirror image differ (A' and B′). (e) A video frame showing a seed of *Ailanthus altissima* in the direct and mirror image (blue and red, respectively). The seed center is represented by two coordinates in the direct image (*x*_d_ and *z*_d_) as well as two coordinates in the mirror image (*y*_m_ and *z*_m_). From these four image coordinates, the presented algorithm reconstructs the seed's three‐dimensional position and estimates the effective *V*_*t*_.

Existing video‐based methods have two main drawbacks: first, they cannot accurately determine a seed’s vertical displacement because they only film the seed from a single perspective. Consequently, a given pixel displacement between two video frames may correspond to different seed displacements, depending on the seed–camera distance (Figure 1a). To account for this, Wyse et al. (2019) estimated the true vertical displacement by recording the landing location of seeds. However, this is labor‐intensive and may still bias *V*_*t*_ estimates when seeds show substantial lateral movement. Secondly, existing video‐based methods fail to account for seed acceleration (Liu et al., 2021) because they estimate *V*_*t*_ as the average velocity in the video (Gómez‐Noguez et al., 2017). This may substantially underestimate *V*_*t*_ for fast‐falling seeds that take longer to approach *V*_*t*_.

Here, we present a new video‐based method that accounts for acceleration and enables nondestructive estimation of falling trajectories and *V*_*t*_ for a broad range of seed types. We demonstrate how to determine the intraspecific variability and repeatability of *V*_*t*_ estimates. We then compare the *V*_*t*_ estimates with measurements of *V*_*t*_ in an existing database.

## 2 DESIGN OF THE APPARATUS

The apparatus consists of a seed release device and a box (Figure S1). The seed release device is a polyvinyl chloride tube (diameter: 10 cm) of adjustable length with an electromagnetic opening flap (Figure S1). When the flap opens, a seed is released and falls through the tube into the box. The box (1.2 × 0.84 × 0.5 m, width × depth × height) contains the falling corridor of 0.25 × 0.25 × 0.33 m (width × depth × height), which is bounded on two adjacent sides by two semitransparent plastic boards that are backlit by two battery‐driven LED lamps to optimize the visual detectability of seeds (Figure 1a,c). To avoid air turbulence due to possible heating, we separated the lamps from the falling corridor with the plastic boards and ensured that the lamps are well ventilated. On the third side of the falling corridor is a high‐speed monochrome camera with a rate of up to 150 frames per second (fps) (acA1920‐155um, Basler AG), 58 cm from the lens to the center of the falling corridor (Figure 1). On the fourth side, a mirror mounted at 62° from the camera plane enables the camera to detect the seed from two perspectives (Figure 1). The camera and opening flap are controlled by the operating software ZR View (developed by Robert Zollner, Munich). When the flap opens, the camera starts recording (in .avi format at 1920 × 1200 pixel resolution; a 2‐s video at 130 fps has a size of 659 MB; Zhu et al., 2021). The apparatus cost 2400 euro in material and 1800 euro in software (June/2016). A single measurement of *V*_*t*_ (including seed release, measurement, and seed collection) takes about 10 s.

## 3 AUTOMATED ESTIMATION OF SEED TRAJECTORIES AND *V*_*T*_

### 3.1 Estimating seed trajectories

In a given image, an object appears both in the direct part of the image (with image coordinates *x*_d_ and *z*_d_) and in the mirror part of the image (with image coordinates *y*_m_ and *z*_m_) (Figure 1e). To reconstruct seed trajectories, these four image coordinates need to be converted into coordinates in three‐dimensional space, where *x* denotes the horizontal position parallel to the camera’s image plane, *y* is the horizontal position in direction of the camera’s optical axis, and *z* is the vertical position (Figure 1). The precise conversion functions depend on the geometry of the apparatus (Figure 1) and image distortion by the mirror and camera. To determine the conversion functions, we marked 35 locations on a 1‐mm grid paper attached to a “calibration board” and placed it upright into the falling corridor at five distances parallel to the camera plane and five distances perpendicular to the camera plane. For each board position, we took a picture with the camera and extracted the direct and mirror coordinates of the 35 marked locations. For each of the two horizontal coordinates (*x* and *y*), we fitted a linear model that predicts the respective coordinate from the main effects and interactions of the two horizontal image coordinates (*x*_d_ and *y*_m_). For the vertical coordinate *z*, we fitted a linear model containing the main effects and interactions of the two vertical image coordinates *z*_d_ and *z*_m_. Each of these three linear conversion models explained >99% of the variance in *x*, *y*, and *z*, respectively. The conversion functions and all other R code are contained in R package “velocimeter” (https://github.com/jinleizhu/velocimeter) developed under R 4.0.3 (R Core Team, 2021).

Videos of falling seeds are analyzed automatically with ImageJ (Schneider et al., 2012). The analysis script (Appendix S1) comprises four steps. First, a stack of inverted images is extracted from each video. Secondly, the first seed‐free image of the stack is subtracted from every image to obtain differential images in which seeds stand out as dark shapes. Thirdly, the differential images are converted to binary images. Fourthly, for all objects above a threshold size, the image coordinates of the object center, as well as the object's area and circularity, are calculated.

R function calculate.terminal.velocity.phys (in package *velocimeter*) then cleans the ImageJ output in four steps. First, it removes objects at the very edges of the falling corridor. Secondly, the largest object in both the direct and the mirror parts of the image is selected. Thirdly, the function considers the resulting pair of objects in the direct and mirror parts as representing a putative seed if their vertical coordinates (*z*_d_ and *z*_m_) are sufficiently close. Fourthly, the function selects the longest sequence of consecutive images containing a putative seed as depicting the seed trajectory. After data cleaning, the function uses the abovementioned conversion functions to derive a time series of three‐dimensional seed coordinates.

### 3.2 Estimating terminal velocity

Estimation of *V*_*t*_ requires fitting a model to the measured seed trajectory. This can be an implicit model (such as when estimating *V*_*t*_ as the velocity at the bottom of the falling corridor; Askew et al., 1997), a phenomenological asymptotic model, a simple or a complex physical model. In the following, we use a simple physical model of vertical free fall with air resistance, which assumes that a seed is a sphere (Taylor, 2005) (for details, see Appendix S2). This model assumes that the falling object is a sphere with a Reynolds number *Re* > 10 (so that drag is a quadratic function of velocity). We note, however, that alternative models can be used to estimate *V*_*t*_. The model predicts the vertical distance traveled over time *t* as

(1) $$z\;\left(t\right)=z\left(0\right)-\frac{{V_{t}}^{2}}{g}\ln\left(\cosh\frac{\mathrm{gt}}{V_{t}}\right),$$

where $z\left(0\right)$ is position at t=0, and *g* is the gravitational acceleration (9.81 m/s^2^). We estimated *V*_*t*_ and $z\left(0\right)$ by fitting this simple physical model to the time series of vertical positions using nonlinear least squares (R function nls).

Observed seed falling trajectories may deviate from the simple physical model because of uncertainty in the determination of seed positions via image analysis or because seeds violate model assumptions. In particular, the simple model ignores effects of low Reynolds numbers, small‐scale turbulence at the seed surface, and vertical forces that may result from seed rotation or horizontal seed movement (Hirata et al., 2011).

To assess how well a given model approximates vertical seed trajectories, we compare model predictions and observations in terms of falling velocity. Specifically, we evaluate the average vertical velocity between two successive images and compare this observed velocity to the corresponding velocity predicted by the model. As an overall measure of discrepancy between predicted and observed velocities, we calculated the root mean squared error (RMSE) for each seed trajectory (function rmse.veloc). When RMSE is high, the function absdiff.veloc can be used to plot these velocity differences against time, indicating which phases of the seed trajectory are poorly approximated by the model. These functions are provided in the R package *velocimeter*.

We used the evaluation functions to assess performance of the simple physical model for seeds of five species with very different morphology (Table S2, Figure S4; *n* = 40 seeds per species). The physical model fitted individual falling trajectories very well (*R* ^2^ > .996 for all seeds; Figure S5a), and the median RMSE falling velocity per species was <0.06 m/s, indicating a good to very good approximation of falling velocities (Figure S6).

An introductory video shows how to use the apparatus, analyze the obtained videos, and estimate *V*_*t*_ (Zhu et al., 2021).

## 4 VALIDATION EXPERIMENTS

**FIGURE 2:**
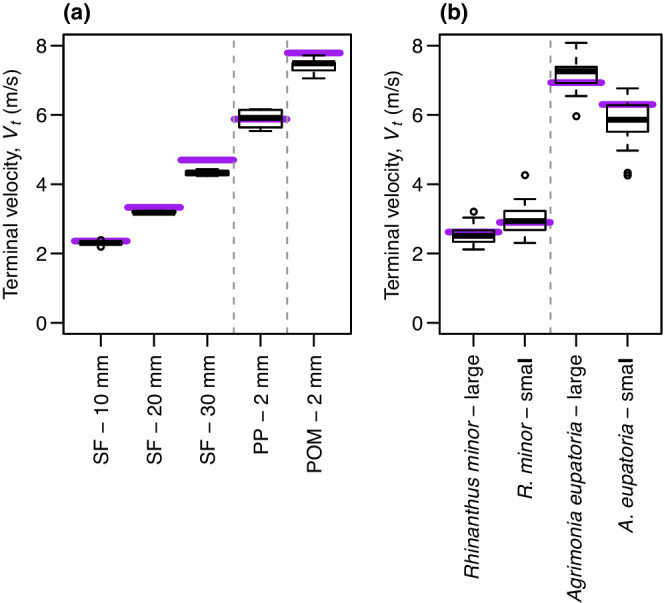
(a) Estimated terminal velocity, *V*_*t*_ (box–whisker plots), and theoretical expectations (horizontal lines) for spheres of different diameters and materials (POM, polyoxymethylene; PP, polypropylene; SF, styrofoam). (b) *V*_*t*_ estimates for seeds in comparison to measurements in a vertical wind tunnel (horizontal lines).

To validate the presented method, we conducted two experiments. In the first experiment, we checked whether our video‐based *V*_*t*_ estimates match theoretical expectations for spheres (Appendix S2). Specifically, we took 15 replicate *V*_*t*_ estimates for each of five sphere types of different diameter *d* and density $\rho_{s}$ (Table S1). These *V*_*t*_ estimates closely match theoretical expectations (Figure 2a; accuracy measured as $1-\left|\mu -E\right|/E$, where μ is mean estimate, and E is expectation: 92.1%–100.0%). The slight underestimation of *V*_*t*_ for spheres made of Styrofoam (30 mm) and Polyoxymethylene might be due to their somewhat rough surface and/or limited applicability of the empirical approximation for the drag coefficient (Appendix S2).

In the second experiment, we compared our video‐based *V*_*t*_ estimates to independent measurements for randomly sampled seeds of *Agrimonia eupatoria* and *Rhinanthus minor* (visually separated into “small” and “large” groups) in a vertical wind tunnel at the Institute of Agricultural Engineering in the Tropics and Subtropics, University of Hohenheim, Germany. The vertical wind tunnel accurately measures the distribution of *V*_*t*_ for seed batches when seed falling trajectories are simple and *V*_*t*_ is relatively high (Karaj & Müller, 2010). We placed batches of 10 seeds per species and size category in the tunnel, gradually increased wind speed (measured with a digital manometer, GDH 01 AN, Greisinger, GHM Messtechnik GmbH), and recorded the wind speed at which the first five seeds were suspended in the air, and calculated *V*_*t*_ following Karaj and Müller (2010). These wind tunnel measurements closely matched our video‐based estimates of *V*_*t*_ (Figure 2b; accuracy: 91.1%–97.1%).

In a third experiment, we showed that *V*_*t*_ estimates are independent of release height (length of the release tube) (Appendix S2).

### 4.1 Intraspecific variability and repeatability of *V*_*t*_ estimates

To determine the intraspecific variability and repeatability of *V*_*t*_ estimates, 10 seeds each of *A. eupatoria*, *Silene vulgaris*, *Iris pseudacorus*, *R*. *minor*, and *Taraxacum officinale* were measured four times. To quantify intraspecific variability, we used a linear mixed‐effects model with log‐transformed *V*_*t*_ as the response variable and seed identity nested within species as the random effect. Variation in *V*_*t*_ was decomposed across the seed and species levels following Messier et al. (2010). To quantify repeatability, we used the intraclass correlation coefficient (ICC) following Wolak et al. (2012).

Across all five study species, species identity explained 98.4% of the variance in log‐transformed terminal velocity, seed identity within species explained 1.0%, and the unexplained variance amounted to 0.6%. The ICC across all seeds of the study species was 0.993 (Table 1), indicating very high repeatability of *V*_*t*_ estimates at this level.

**TABLE 1:** Repeatability of *V*_*t*_ estimates for individual seeds, estimated as the intraclass correlation coefficient (ICC) following Wolak et al. (2012).

Species/seeds	ICC
All species	.993
*Agrimonia eupatoria*	.698
*Silene vulgaris*	.550
*Iris pseudacorus*	.372
*Rhinanthus minor*	.208
*Taraxacum officinale*	.887

Within individual species, seed identity explained between 39% and 91% (median 63%) of the variance in log‐transformed terminal velocity, with the remaining proportion arising from the variance between repeated measures of the same seed (Table 2). The ICC ranged from 0.208 for *R. minor* to 0.887 for *T. officinale* (median 0.550) (Table 1). The low repeatability of seed‐level *V*_*t*_ estimates in *R. minor* might in principle result solely from measurement error. Yet it is likely to be at least partly caused by the fact that seeds of this species show variable orientation in replicate experimental drops (Figure S7).

**TABLE 2:** Intraspecific variability of the measured seed terminal velocity (*V*_*t*_) within individual species. Each of the 10 seeds of each species was measured four times. Intraspecific variability was quantified as the coefficient of determination (*R* ^2^) of the linear model in which log‐transformed *V*_*t*_ was the response variable, and seed identity was the explanatory variable.

Species	*R* ^2^
*Agrimonia eupatoria*	.76
*Silene vulgaris*	.63
*Iris pseudacorus*	.51
*Rhinanthus minor*	.39
*Taraxacum officinale*	.91

### 4.2 Comparison to existing *V*_*t*_ measurements

**FIGURE 3:**
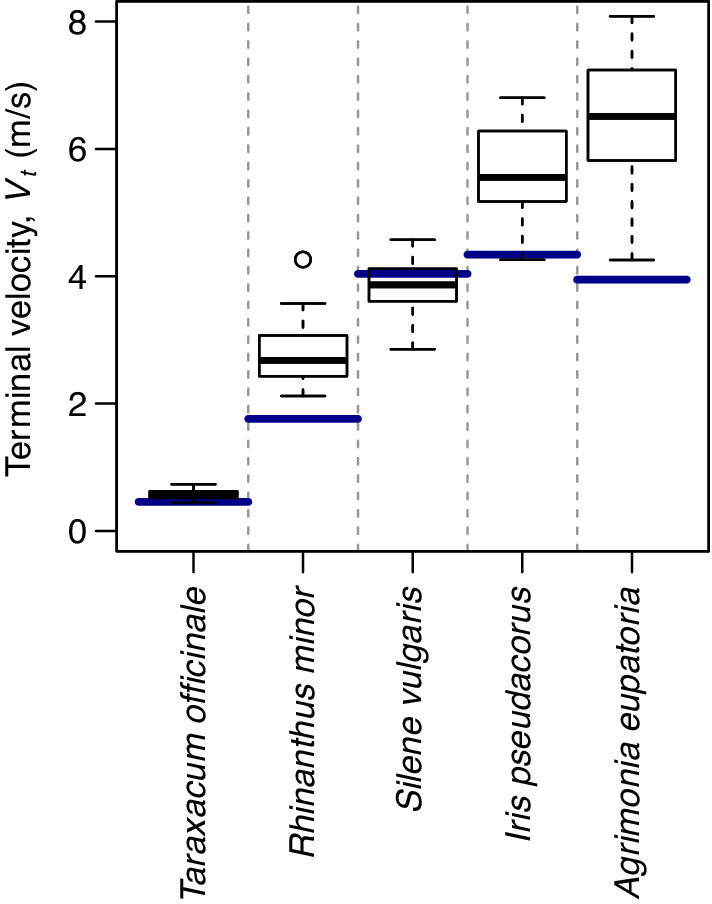
The estimated terminal velocity of seeds in comparison to mean values (blue horizontal lines) in the TRY database (Kattge et al., 2020).

We compared our *V*_*t*_ estimates for five plant species that cover a variety of seed sizes and shapes to the mean *V*_*t*_ in the TRY database, which predominantly contains measurements of average velocity after seeds had fallen a certain distance and thus does not account for seed acceleration (Kattge et al., 2020). For four out of these five species, the TRY values are substantially lower than our *V*_*t*_ estimates (Figure 3). As expected, the underestimation of *V*_*t*_ in the TRY database is more pronounced for fast‐falling seeds that take longer to approach *V*_*t*_ (Figure S3).

## 5 DISCUSSION

The presented method for quantifying seed falling trajectories and estimating *V*_*t*_ has six main advantages over previous methods. First, the method can quantify seed acceleration. Secondly, the method can estimate the falling trajectory (Figure S2) and *V*_*t*_ of single seeds in a nondestructive manner. Thirdly, our method improves on existing video‐based methods that can produce erroneous estimates of *V*_*t*_ by not accounting for the distance between the camera and the falling seed (Wyse et al., 2019). To accurately determine the vertical position of a falling seed, one needs to film the seed from two perspectives simultaneously. This could be achieved by using two cameras, but this is costly and raises the challenge of accurately synchronizing the cameras. Fourthly, the simple physical model accurately estimated *V*_*t*_ of seeds that were still accelerating considerably (Figure S5). This can greatly increase time efficiency because one does not have to ensure that a falling seed is close to *V*_*t*_ in the falling corridor (Sullivan et al., 2018). It also substantially increases practicability: large *A. eupatoria* seeds (mean *V*_*t*_ 6.4 m/s) only reach 99% of *V*_*t*_ after a falling distance of 8.2 m (Figure S3). This clearly exceeds the dimensions of most ecological laboratories. Fifthly, the evaluation functions enable users to assess the validity of the model used to estimate *V*_*t*_ from falling trajectories. To our knowledge, no other method permits assessing the validity of *V*_*t*_ estimates. Lastly, the method is highly automated and can rapidly estimate the *V*_*t*_ of large numbers of seeds. While existing methods should yield reliable estimates of *V*_*t*_ for many slow‐falling seeds, the new method presented here has a greater scope of application and provides additional insights into the mechanisms of wind‐driven seed dispersal. We discuss these aspects in the following.

### 5.1 Applicability of the method to different seed sizes

We successfully estimated *V*_*t*_ of seeds ranging from 0.7 mm to 3 cm in diameter. Yet our apparatus cannot be used for very large and very small seeds. However, the method is in principle applicable to seeds of any size. Larger seeds can be accommodated by increasing the dimensions of the apparatus, whereas smaller seeds can be estimated by reducing these dimensions and/or using a higher‐resolution camera.

### 5.2 Improving the quantification of seed dispersal

The presented method has important implications for the quantification of wind‐driven seed dispersal. The first implication is that many *V*_*t*_ estimates in existing databases (e.g. TRY; Kattge et al., 2020) probably underestimate the true *V*_*t*_ of fast‐falling seeds (Figure 3). Such underestimation results if *V*_*t*_ is taken to be the average falling velocity after a limited acceleration distance that is insufficient for the seed to approach *V*_*t*_ (Figure 2). This underestimation of *V*_*t*_ should cause mechanistic dispersal models to overestimate distances of wind‐driven seed dispersal. It could be argued that this overestimation is ecologically irrelevant because fast‐falling seeds invariably have short dispersal distances. However, the mechanistic model of Nathan et al. (2002) predicts that strong uplifts can even transport hickory nuts (*Carya glabra*), which have a very high *V*_*t*_ (7.84 m/s), over several hundred meters (Higgins et al., 2003). Since the uplift probability depends on *V*_*t*_, reliable mechanistic predictions of dispersal distance require accurate *V*_*t*_ estimates even for fast‐falling seeds.

A second implication of underestimating *V*_*t*_ is an underestimation of seed inertia, as characterized by the Lagrangian relaxation timescale τ (Equation S6). Seeds with high τ take longer to accelerate in response to gravity or changes in vertical and horizontal wind speed (Nathan et al., 2011). According to the simple physical model fitted to our data for *A. eupatoria*, an average seed that drops in still air from the species' average seed release height (0.45 m; Kleyer et al., 2008) has reached only 44% of *V*_*t*_ when it hits the ground. Mechanistic models that ignore seed inertia and assume that seeds fall at *V*_*t*_ right after seed release will thus underestimate dispersal ability, in particular for smaller plants with fast‐falling seeds. However, seed inertia is not represented by most mechanistic models for wind dispersal (Nathan et al., 2011) including the widely used WALD model (Katul et al., 2005; for exceptions see Bohrer et al., 2008; Kuparinen et al., 2007). Our method provides data on seed falling trajectories that can be used to validate, inversely parameterize, and/or select between models of seed acceleration. Candidate models are the simple physical model used here or more complex models that represent effects of nonspherical seed morphologies, nonquadratic drag, seed rotation, and others (Hirata et al., 2011; Lentink et al., 2009; Schwendemann et al., 2007). Suitable acceleration models can then be incorporated into improved mechanistic models for wind‐driven seed dispersal.

A third implication of the presented method is that it should advance quantification of the causes and consequences of intraspecific variation in seed dispersal (Albert et al., 2011; Chen & Giladi, 2020; Snell et al., 2019; Zhu et al., 2019). In species with low seed‐level repeatability of falling trajectories and *V*_*t*_ (such as *R. minor*), the video‐based method can be used to quantify to what extent within‐seed variability arises from measurement error versus intrinsic variability in the flight behavior of a given seed (Figure S3). This decomposition of variability in seed dispersal has important consequences for understanding the evolution of seed dispersal: the larger the intrinsic component, the less dispersal is controlled by seed traits and hence the genotype (Schurr et al., 2009). In species with high seed‐level repeatability of falling trajectories and *V*_*t*_ (such as *T. officinale*), the presented method should enable the efficient dispersal phenotyping of seeds from multiple plant genotypes grown in multiple environments. This would be a crucial step toward unraveling the genetic architecture of how dispersal responds to environmental variation and quantifying how rapidly dispersal can evolve in changing environments (Ronce, 2007).

## 6 CONCLUSIONS

We present a novel video‐based method that determines the three‐dimensional trajectory of falling seeds and analyzes these trajectories with a simple physical model of free fall with air resistance to estimate *V*_*t*_. This accurate, efficient, and affordable method improves the quantification of intra‐ and interspecific variation in seed dispersal ability and opens new avenues for dispersal research. It should thus be an important addition to the toolbox of plant biologists.

## AUTHOR CONTRIBUTIONS

**Jinlei Zhu:** Conceptualization (lead); data curation (lead); formal analysis (lead); methodology (lead); software (lead); visualization (lead); writing – original draft (lead); writing – review and editing (lead). **Carsten M. Buchmann:** Conceptualization (lead); data curation (supporting); formal analysis (supporting); methodology (lead); software (lead); validation (lead); visualization (supporting); writing – original draft (supporting); writing – review and editing (supporting). **Frank M. Schurr:** Conceptualization (lead); funding acquisition (lead); methodology (supporting); project administration (equal); software (supporting); supervision (lead); visualization (supporting); writing – original draft (supporting); writing – review and editing (supporting).

## CONFLICT OF INTEREST

The authors have no conflict of interest.

## Supporting information


Supporting Information

## Data Availability

Data on seed terminal velocity are archived in the Dryad Digital Repository (https://doi.org/10.5061/dryad.gf1vhhms6; Zhu et al., 2022). The ImageJ script is in the supporting information. R code is available at https://github.com/jinleizhu/velocimeter.

## References

[ece39183-bib-0001] Albert, C. H. , Grassein, F. , Schurr, F. M. , Vieilledent, G. , & Violle, C. (2011). When and how should intraspecific variability be considered in trait‐based plant ecology? Perspectives in Plant Ecology, Evolution and Systematics, 13(3), 217–225. 10.1016/j.ppees.2011.04.003

[ece39183-bib-0002] Askew, A. P. , Corker, D. , Hodkinson, D. J. , & Thompson, K. (1997). A new apparatus to measure the rate of fall of seeds. Functional Ecology, 11(1), 121–125. 10.1046/j.1365-2435.1997.00049.x

[ece39183-bib-0003] Bohrer, G. , Katul, G. G. , Nathan, R. , Walko, R. L. , & Avissar, R. (2008). Effects of canopy heterogeneity, seed abscission and inertia on wind‐driven dispersal kernels of tree seeds. Journal of Ecology, 96(4), 569–580. 10.1111/j.1365-2745.2008.01368.x

[ece39183-bib-0004] Chen, S. C. , & Giladi, I. (2020). Variation in morphological traits affects dispersal and seedling emergence in dispersive diaspores of *Geropogon hybridus* . American Journal of Botany, 107(3), 436–444. 10.1002/ajb2.1430 32072626 PMC7154696

[ece39183-bib-0005] Gómez‐Noguez, F. , León‐Rossano, L. M. , Mehltreter, K. , Orozco‐Segovia, A. , Rosas‐Pérez, I. , & Pérez‐García, B. (2017). Experimental measurements of terminal velocity of fern spores. American Fern Journal, 107(2), 59–71. 10.1640/0002-8444-107.2.59

[ece39183-bib-0006] Higgins, S. I. , Nathan, R. , & Cain, M. L. (2003). Are long‐distance dispersal events in plants usually caused by nonstandard means of dispersal? Ecology, 84(8), 1945–1956. 10.1890/01-0616

[ece39183-bib-0007] Hirata, K. , Hayakawa, M. , & Funaki, J. (2011). On tumbling of a two‐dimensional plate under free flight. Journal of Fluid Science and Technology, 6(2), 177–191. 10.1299/jfst.6.177

[ece39183-bib-0008] Jongejans, E. , & Schippers, P. (1999). Modeling seed dispersal by wind in herbaceous species. Oikos, 87(2), 362. 10.2307/3546752

[ece39183-bib-0009] Karaj, S. , & Müller, J. (2010). Determination of physical, mechanical and chemical properties of seeds and kernels of *Jatropha curcas* L. Industrial Crops and Products, 32(2), 129–138. 10.1016/j.indcrop.2010.04.001

[ece39183-bib-0010] Kattge, J. , Bönisch, G. , Díaz, S. , Lavorel, S. , Prentice, I. C. , Leadley, P. , Tautenhahn, S. , Werner, G. D. A. , Aakala, T. , Abedi, M. , Acosta, A. T. R. , Adamidis, G. C. , Adamson, K. , Aiba, M. , Albert, C. H. , Alcántara, J. M. , Alcázar, C. C. , Aleixo, I. , Ali, H. , … Wirth, C. (2020). TRY plant trait database – Enhanced coverage and open access. Global Change Biology, 26(1), 119–188. 10.1111/gcb.14904 31891233

[ece39183-bib-0011] Katul, G. G. , Porporato, A. , Nathan, R. , Siqueira, M. , Soons, M. B. , Poggi, D. , Horn, H. S. , & Levin, S. A. (2005). Mechanistic analytical models for long‐distance seed dispersal by wind. American Naturalist, 166(3), 368–381. 10.1086/432589 16224691

[ece39183-bib-0012] Kleyer, M. , Bekker, R. M. , Knevel, I. C. , Bakker, J. P. , Thompson, K. , Sonnenschein, M. , Poschlod, P. , Van Groenendael, J. M. , Klimeš, L. , Klimešová, J. , Klotz, S. , Rusch, G. M. , Hermy, M. , Adriaens, D. , Boedeltje, G. , Bossuyt, B. , Dannemann, A. , Endels, P. , Götzenberger, L. , … Peco, B. (2008). The LEDA Traitbase: A database of life‐history traits of the northwest European flora. Journal of Ecology, 96(6), 1266–1274. 10.1111/j.1365-2745.2008.01430.x

[ece39183-bib-0013] Kuparinen, A. , Markkanen, T. , Riikonen, H. , & Vesala, T. (2007). Modeling air‐mediated dispersal of spores, pollen and seeds in forested areas. Ecological Modelling, 208(2–4), 177–188. 10.1016/j.ecolmodel.2007.05.023

[ece39183-bib-0014] Lentink, D. , Dickson, W. B. , Van Leeuwen, J. L. , & Dickinson, M. H. (2009). Leading‐edge vortices elevate lift of autorotating plant seeds. Science, 324(5933), 1438–1440. 10.1126/science.1174196 19520959

[ece39183-bib-0015] Liu, M. , Xin, Z. , Su, Z. , Zhao, Y. , Li, X. , Liu, Z. , Cony, M. A. , Liang, W. , Qin, X. , Qian, J. , Cui, X. , & Zhou, Q. (2021). A video camera recording method for measuring terminal velocity of seed dispersal by wind. Journal of Forestry Research, 32(1), 81–90. 10.1007/s11676-019-01092-8

[ece39183-bib-0016] Loubet, B. , Jarosz, N. , Saint‐Jean, S. , & Huber, L. (2007). A method for measuring the settling velocity distribution of large biotic particles. Aerobiologia, 23(3), 159–169. 10.1007/s10453-007-9054-2

[ece39183-bib-0017] Messier, J. , McGill, B. J. , & Lechowicz, M. J. (2010). How do traits vary across ecological scales? A case for trait‐based ecology. Ecology Letters, 13(7), 838–848. 10.1111/j.1461-0248.2010.01476.x 20482582

[ece39183-bib-0018] Nathan, R. , Katul, G. G. , Bohrer, G. , Kuparinen, A. , Soons, M. B. , Thompson, S. E. , Trakhtenbrot, A. , & Horn, H. S. (2011). Mechanistic models of seed dispersal by wind. Theoretical Ecology, 4(2), 113–132. 10.1007/s12080-011-0115-3

[ece39183-bib-0019] Nathan, R. , Katul, G. G. , Horn, H. S. , Thomas, S. M. , Oren, R. , Avissar, R. , Pacala, S. W. , & Levin, S. A. (2002). Mechanisms of long‐distance dispersal of seeds by wind. Nature, 418(6896), 409–413. 10.1038/nature00844 12140556

[ece39183-bib-0020] R Core Team . (2021). R: A language and environment for statistical computing. R Foundation for Statistical Computing. https://www.R‐project.org/

[ece39183-bib-0021] Ronce, O. (2007). How does it feel to be like a rolling stone? Ten questions about dispersal evolution. Annual Review of Ecology, Evolution, and Systematics, 38, 231–253. 10.1146/annurev.ecolsys.38.091206.095611

[ece39183-bib-0022] Russo, G. P. (2011). Aerodynamic measurements: From physical principles to turnkey instrumentation. Woodhead Publishing Limited.

[ece39183-bib-0023] Schneider, C. A. , Rasband, W. S. , & Eliceiri, K. W. (2012). NIH image to ImageJ: 25 years of image analysis. Nature Methods, 9(7), 671–675. 10.1038/nmeth.2089 22930834 PMC5554542

[ece39183-bib-0024] Schurr, F. M. , Spiegel, O. , Steinitz, O. , Trakhtenbrot, A. , Tsoar, A. , & Nathan, R. (2009). Long‐distance seed dispersal. In Fruit development and seed dispersal (pp. 204–237). Wiley‐Blackwell. 10.1002/9781444314557.ch6 18823680

[ece39183-bib-0025] Schwendemann, A. B. , Wang, G. , Mertz, M. L. , McWilliams, R. T. , Thatcher, S. L. , & Osborn, J. M. (2007). Aerodynamics of saccate pollen and its implications for wind pollination. American Journal of Botany, 94(8), 1371–1381. 10.3732/ajb.94.8.1371 21636505

[ece39183-bib-0026] Snell, R. S. , Beckman, N. G. , Fricke, E. , Loiselle, B. A. , Carvalho, C. S. , Jones, L. R. , Lichti, N. I. , Lustenhouwer, N. , Schreiber, S. J. , Strickland, C. , Sullivan, L. L. , Cavazos, B. R. , Giladi, I. , Hastings, A. , Holbrook, K. M. , Jongejans, E. , Kogan, O. , Montaño‐Centellas, F. , Rudolph, J. , … Schupp, E. W. (2019). Consequences of intraspecific variation in seed dispersal for plant demography, communities, evolution and global change. AoB PLANTS, 11(4), plz016. 10.1093/aobpla/plz016 31346404 PMC6644487

[ece39183-bib-0027] Sullivan, L. L. , Clark, A. T. , Tilman, D. , & Shaw, A. K. (2018). Mechanistically derived dispersal kernels explain species‐level patterns of recruitment and succession. Ecology, 99(11), 2415–2420. 10.1002/ecy.2498 30368793

[ece39183-bib-0028] Taylor, J. R. (2005). Classical mechanics. University Science Books.

[ece39183-bib-0029] Wolak, M. E. , Fairbairn, D. J. , & Paulsen, Y. R. (2012). Guidelines for estimating repeatability. Methods in Ecology and Evolution, 3(1), 129–137. 10.1111/j.2041-210X.2011.00125.x

[ece39183-bib-0030] Wyse, S. V. , Hulme, P. E. , & Holland, E. P. (2019). Partitioning intraspecific variation in seed dispersal potential using a low‐cost method for rapid estimation of samara terminal velocity. Methods in Ecology and Evolution, 10(8), 1298–1307. 10.1111/2041-210X.13202

[ece39183-bib-0031] Zhu, J. , Buchmann, C. , & Schurr, F. (2022). Improving measurements of the falling trajectory and terminal velocity of wind‐dispersed seeds. Dryad. Dataset. 10.5061/dryad.gf1vhhms6 PMC9353119 35949535

[ece39183-bib-0032] Zhu, J. , Buchmann, C. , & Schurr, F. M. (2021). Introductory video of velocimeter and videos of seed falling. figshare. Media. 10.6084/m9.figshare.16575011.v2

[ece39183-bib-0033] Zhu, J. , Liu, M. , Xin, Z. , Liu, Z. , & Schurr, F. M. (2019). A trade‐off between primary and secondary seed dispersal by wind. Plant Ecology, 220(4–5), 541–552. 10.1007/s11258-019-00934-z

