# Supplementary material for: Improving measurements of the falling trajectory and terminal velocity of wind‐dispersed seeds
Source: Ecol Evol. 2022 Aug 4;12(8):e9183. doi: 10.1002/ece3.9183 (PMC9353119; doi:10.1002/ece3.9183)
Supplement: Supplementary file 1 — Supporting Information [file ECE3-12-e9183-s001.docx]

Supporting Information for

**Improving measurements of the falling trajectory and terminal velocity of wind-dispersed seeds**

Jinlei Zhu^*, †^, Carsten M. Buchmann^†^, Frank M. Schurr

Institute of Landscape and Plant Ecology, University of Hohenheim, 70599 Stuttgart, Germany

* Corresponding author: [jinlei.zhu@uni-hohenheim.de](mailto:jinlei.zhu@uni-hohenheim.de)

^†^ These authors made equal contributions to this work.

ORCiD IDs:

Jinlei Zhu: <http://orcid.org/0000-0001-6164-0472>

Carsten M. Buchmann: <https://orcid.org/0000-0002-9926-051X>

Frank M. Schurr: <https://orcid.org/0000-0002-4518-0602>


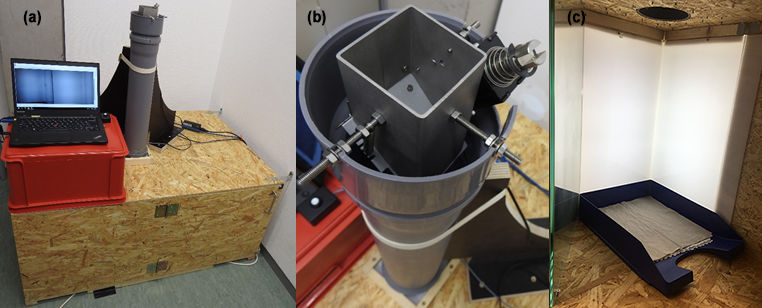


**Fig. S1.** Construction of the apparatus for measuring terminal seed falling velocity. (a) Overall view, (b) electromagnetic release flap, and (c) falling corridor with the illuminated front and side boards, the mirror on the left side and a tray to collect seeds.

**Table S1.** Spheres used in the validation experiment, and their theoretical *V_t_* values calculated according to eqn. S7 (Cheng, 2009).

| Diameter (mm) | Material | Density (kg/m^3^) | Theoretical *V_t_* (m/s) |
| --- | --- | --- | --- |
| 2.0 | Polyoxymethylene (POM)^*^ | 1265.3 | 8.04 |
| 2.0 | Polypropylene (PP)^*^ | 859.4 | 6.49 |
| 10.2 | Styrofoam (SF)^**^ | 21.6 | 2.40 |
| 20.2 | Styrofoam (SF) | 20.9 | 3.39 |
| 28.7 | Styrofoam (SF) | 30.0 | 4.80 |

*Supplier: IHSD-Klarmann, Bamberg, Germany; **Supplier: Meyercordt GmbH, Bad Salzuflen, Germany


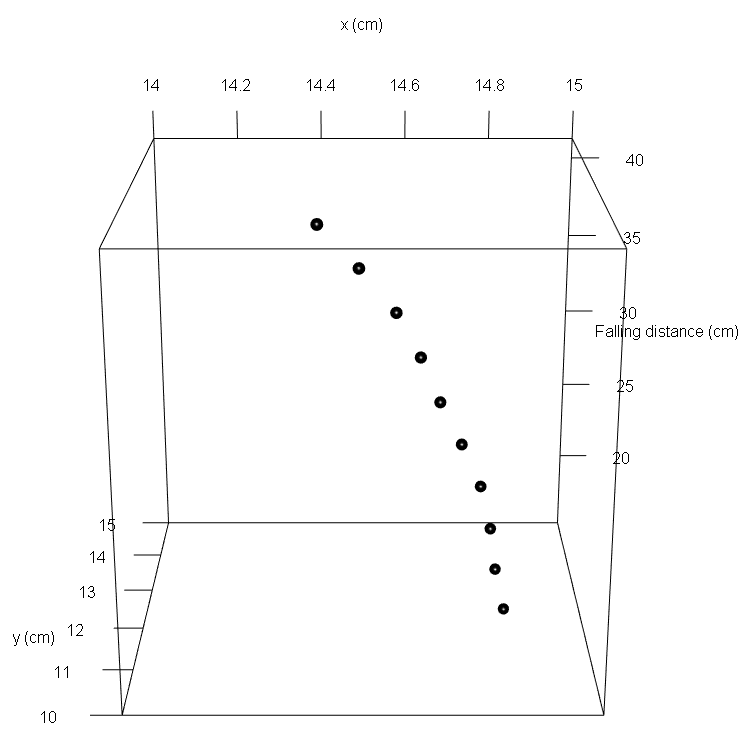


**Fig. S2**. 3D-trajectory of a falling seed of *Agrimonia eupatoria*.

**Appendix 1:** ImageJ script to automatically analyse the videos of falling seeds.

function getNumericTag(tag) {

value = getTag(tag);

if (value=="") return NaN;

index3 = indexOf(value, " ");

if (index3>0)

value = substring(value, 0, index3);

value = 0 + value; // convert to number

return value;

}

function getTag(tag) {

info = getImageInfo();

index1 = indexOf(info, tag);

if (index1==-1) return "";

index1 = indexOf(info, ":", index1);

if (index1==-1) return "";

index2 = indexOf(info, "\n", index1);

value = substring(info, index1+1, index2);

return value;

}

dir = getDirectory("Choose a Directory ");

list = getFileList(dir);

getDateAndTime(year, month, week, day, hour, min, sec, msec);

outdir = dir+"terminal_velocity_analysis"+toString(year)+toString(month)+toString(day)+toString(hour)+toString(min);

File.makeDirectory(outdir)

for (i=0; i<list.length; i++) {

if (!endsWith(list[i], "/"))

{

open(list[i]);

run("Invert", "stack");

rename("0");

run("Make Substack...", " slices=1");

rename("1");

imageCalculator("Subtract create stack", "0","1");

doCommand("Start Animation [\\]");

setAutoThreshold("Default");

//run("Threshold...");

setAutoThreshold("Default dark");

run("Convert to Mask", "method=Default background=Default");

run("Make Binary", "method=Default background=Default");

run("Set Measurements...", "area center shape stack redirect=None decimal=3");

run("Analyze Particles...", "size=20-Infinity display stack");

saveAs("Measurements", outdir+"/"+list[i]+"Results.txt");

close(list[i]);

run("Close All");

run("Clear Results");

}

}

**Appendix 2:** Details on the physical model and the third validation experiment.

*Physical model for free fall with air resistance*

Estimation of *V_t_* requires fitting a model to the measured seed trajectory. This can be an implicit model (such as when estimating *V_t_* as the velocity at the bottom of the falling corridor (Askew, Corker, Hodkinson, & Thompson, 1997)), a phenomenological asymptotic model, a simple or a complex physical model. In the following, we use a simple physical model of vertical free fall with air resistance which assumes that a seed is a sphere. The vertical motion of such a sphere falling in still air is described by

$m a_{z}\left( t \right)=m {v_{z}}^{'}\left( t \right)=F_{G}$ - $F_{D}\left( t \right)$, (eqn. S1)

where $m$ is sphere mass, $a_{z}(t)$ is downward acceleration as a function of time $t$, ${v_{z}}^{'}\left( t \right)$ is the derivative of falling velocity $v_{z}$ with respect to time, and $F_{G}=mg$ is the absolute value of gravitational force (with gravitational acceleration $g$ = 9.81 m/s^2^). $F_{D}\left( t \right)$ is the time-dependent absolute value of the aerodynamic drag force, determined by

$F_{D}\left( t \right)=\frac{1}{2}\rho C_{D}A{v_{z}(t)}^{2}$, (eqn. S2)

where $\rho$ is the air density, $A$ is the planform area of the sphere and $C_{D}$ is the sphere’s drag coefficient. For objects with sufficiently large Reynolds numbers (Re), including most seeds (Re > 10) (Nathan et al., 2011), $C_{D}$ is essentially constant (Vogel, 1994). In the following, we thus assume that drag $F_{D}\left( t \right)$ is proportional to ${v_{z}(t)}^{2}$ (so-called ‘quadratic drag’). However, time series of vertical coordinates produced by our apparatus could also be used to estimate *V_t_* for dust-like seeds and pollen (Re < 1) for which $F_{D}\left( t \right)$ is proportional to $v_{z}\left( t \right)$ (‘linear drag’) and for propagules in the transition zone between linear and quadratic drag.

Assuming quadratic drag and a sphere starting from rest ($v_{z}\left( t \right)=0$), one can substitute eqn. S2 into eqn. S1, solve for ${v_{z}}^{'}\left( t \right)$ and integrate over time $t$ to obtain an equation for the time-dependent sphere falling velocity

$v_{z}\left( t \right)=V_{t}\tanh\frac{gt}{V_{t}}$, (eqn. S3)

with terminal velocity

$V_{t}=\sqrt{\frac{2mg}{\rho C_{D}A}}$, (eqn. S4)

Further integration of eqn. S3 over time $t$ leads to a time-dependent expression for the vertical distance travelled by the falling sphere, $z \left( t \right)$

$z \left( t \right)=z\left( 0 \right)-\frac{{V_{t}}^{2}}{g}\ln\left( \cosh\frac{gt}{V_{t}} \right)$, (eqn. S5; (Taylor, 2005))

where $z\left( 0 \right)$ is sphere position at $t=0$ (in the presented apparatus this may differ from 0 due to dislocation by the opening flap). We estimated *V_t_* and $z\left( 0 \right)$ by fitting this physical model of free fall with air resistance to the time series of vertical sphere position (using nonlinear least squares, R function nls).

Note that from eqn. S3 one can derive the Lagrangian relaxation timescale τ, the time a sphere falling from rest takes to reach a high proportion $p$ of *V_t_*

$\tau=\frac{V_{t}}{g}\mathrm{atanh} p$, (eqn. S6)

τ characterizes the consequences of seed inertia for dispersal (Nathan et al., 2011). Inserting τ into eqn. S5 yields the falling distance *z*(τ) that a sphere needs to approach *V_t_*. This "acceleration distance" strongly increases with *V_t_* and can be considerable for fast-falling spheres (Fig. S3).

**Fig. S3.** Relationship between a sphere’s terminal falling velocity (*V_t_*) and the distance over which the sphere has to fall to reach a certain proportion $p$ of *V_t_*. Note that this relationship holds for large Reynolds numbers (Re > 10).

*Theoretically expected V_t_ of spheres*

Eqn. S4 can be used to calculate the theoretically expected *V_t_* for spheres with diameter *d* (so that $A=\pi/{4d^{2}})$and density $\rho_{s}$ (Table S1).

Specifically, we set air density to $\rho$ =1.20 kg/m^3^ (at 20°C and 1 atm), and

$C_{D}=\frac{432}{d_{*}^{3}}\left( 1+0.022d_{*}^{3} \right)^{0.54}+\left[ 1-exp\left( -0.15d_{*}^{0.45} \right) \right]$, (eqn. S7; (Cheng, 2009))

with $d_{*}=\left( \frac{\left( \rho_{s}-\rho\right)g}{\rho\nu} \right)^{1/3}$, (eqn. S8; (Cheng, 2009))

where ν is the kinematic viscosity of air (15.06 x 10^-6^ m^2^/s at 20°C and 1 atm).

*Seed trajectories and estimates of terminal velocity are independent of release height*

We examined whether estimated seed trajectories and terminal velocities are independent of seed release height. To this end, we measured seeds of four plant species with different morphologies (*Agrimonia eupatoria*, *Silene vulgaris, Iris pseudacorus*, and *Rhinanthus minor*) (Table S2; Fig. S4) for each of three release heights (2095 mm, 1575 mm, and 1075 mm distance from the bottom of the release flap to the bottom of the falling corridor). These different release heights were achieved by inserting tubes of different lengths between the release flap and the falling corridor. For each combination of release height and species, we measured 30 seeds, distinguishing between 15 smaller and 15 larger seeds to account for intraspecific variation in seed properties.

**Table S2.** Seed properties of the study species. Seed mass was calculated as the average mass of 15 seeds per species and size class (30 seeds in the case of *Taraxacum*). Terminal velocity (*V_t_*) was acquired from the TRY database (Kattge et al., 2020), and is expressed as mean ± SD.

| Species | Seed morphology | Seed mass (g) | | *V_t_* in TRY (m/s) |
| --- | --- | --- | --- | --- |
|  |  | large | small |  |
| *Agrimonia eupatoria* | Spherical, hooked | 0.0260 | 0.0100 | 3.95 ± 0.32 |
| *Silene vulgaris* | Spherical | 0.0008 | 0.0006 | 4.04 ± 0.22 |
| *Iris pseudacorus* | Disk | 0.0486 | 0.0388 | 4.34 ± 0.24 |
| *Rhinanthus minor* | Flat disk with wings | 0.0040 | 0.0038 | 1.76 ± 0.26 |
| *Taraxacum officinale* | Pappus | 0.0005^*^ | | 0.46 ± 0.16 |

*Average seed mass of 30 seeds without being separated into large and small groups.


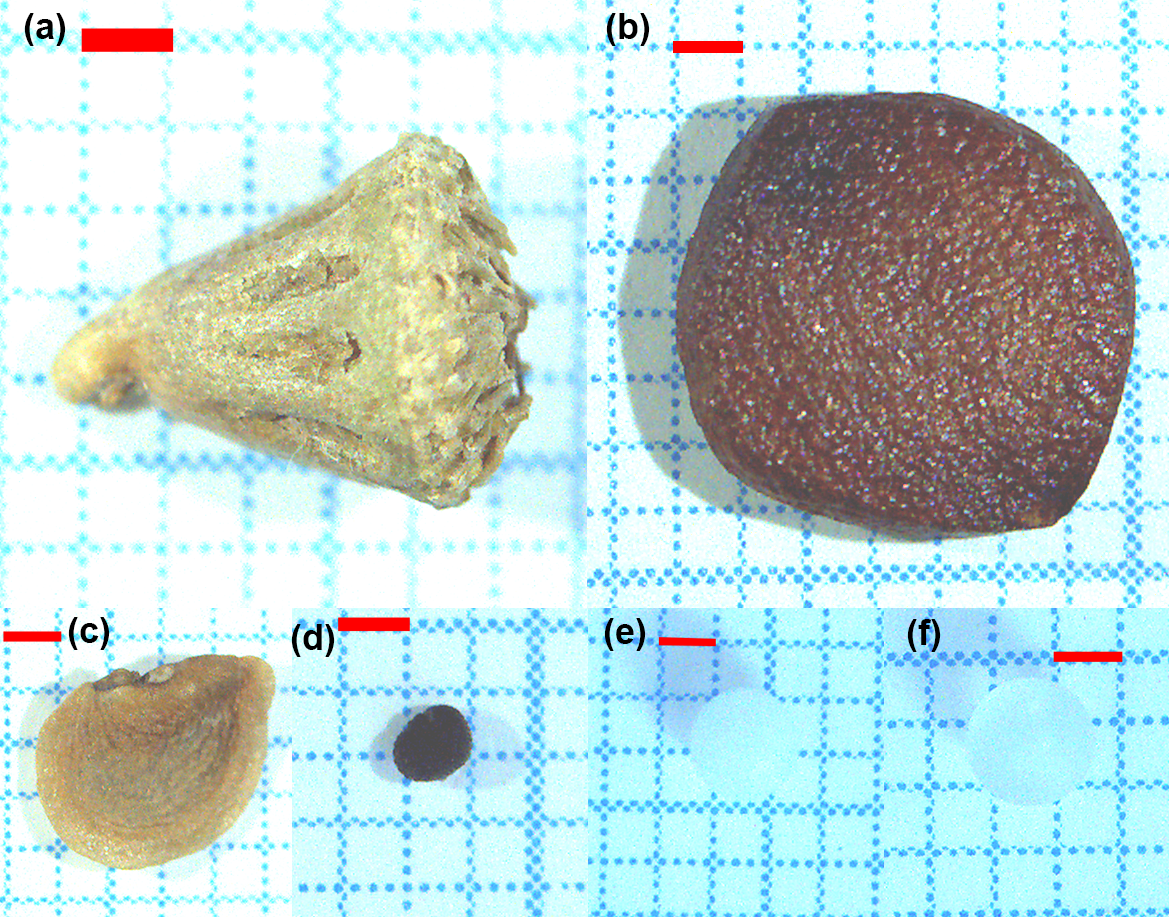


**Fig. S4.** Seeds and spheres used in the study. Seeds of (a) *Agrimonia eupatoria*, (b) *Iris pseudacorus*, (c) *Rhinanthus minor*, (d) *Silene vulgaris*. Spheres made of (e) Polyoxymethylene and (f) Polypropylene. Red bars indicate a length of 1 mm.

The presented video-based method estimated very similar seed falling trajectories when the same seed type was released from different heights (Fig. S5a). Accordingly, the physical model yields estimates of terminal seed falling velocity (*V_t_*) that are independent of seed release height (Fig. S5b).

The recorded trajectories of individual seeds in the falling corridor often show an almost linear relationship between vertical position and time (insert in Fig. S5a). This might be taken to indicate that seeds do not accelerate anymore and that *V_t_* can simply be estimated as the average seed velocity in the falling corridor (as in Askew et al. (1997) and similar approaches). We thus calculated this average seed velocity as the slope of a linear regression of vertical position against time. Yet for fast-falling seeds (large and small *A. eupatoria*), the average velocity increased strongly with release height and was consistently lower than the *V_t_* estimated with the physical model (Fig. S5b). In contrast, for slow-falling seeds (small and large *R. minor*) the average velocity was largely independent of release height and matched *V_t_* estimates of the physical model (Fig. S5b). This indicates that the fast-falling seeds of *A. eupatoria* still accelerated considerably even when released from > 2 m height.

**
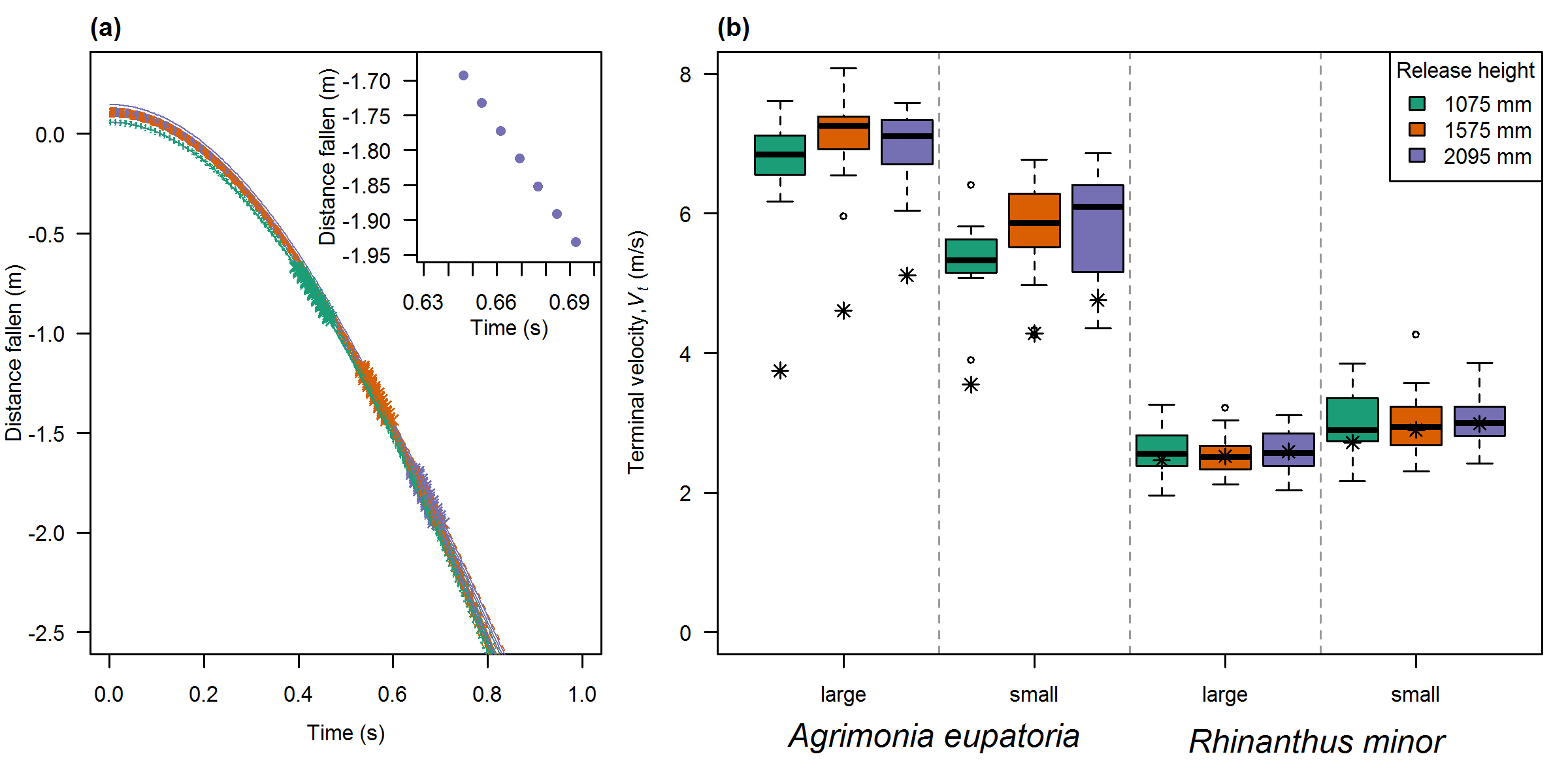
**

**Fig. S5.** (a) Relationship between distance fallen (vertical displacement) and time for large seeds of *Agrimonia eupatoria* released from different heights (colours). The lines represent predictions of a physical model of free fall with air resistance fitted to the respective data set. The insert represents the relationship between distance fallen and time for a single large seed of *A. eupatoria* released from the height of 2095 mm. (b) Box-whisker plots showing terminal velocity estimates of the physical model for seeds of two plant species and two sizes that were released from different heights. Asterisks indicate the recorded average velocity in the falling corridor.


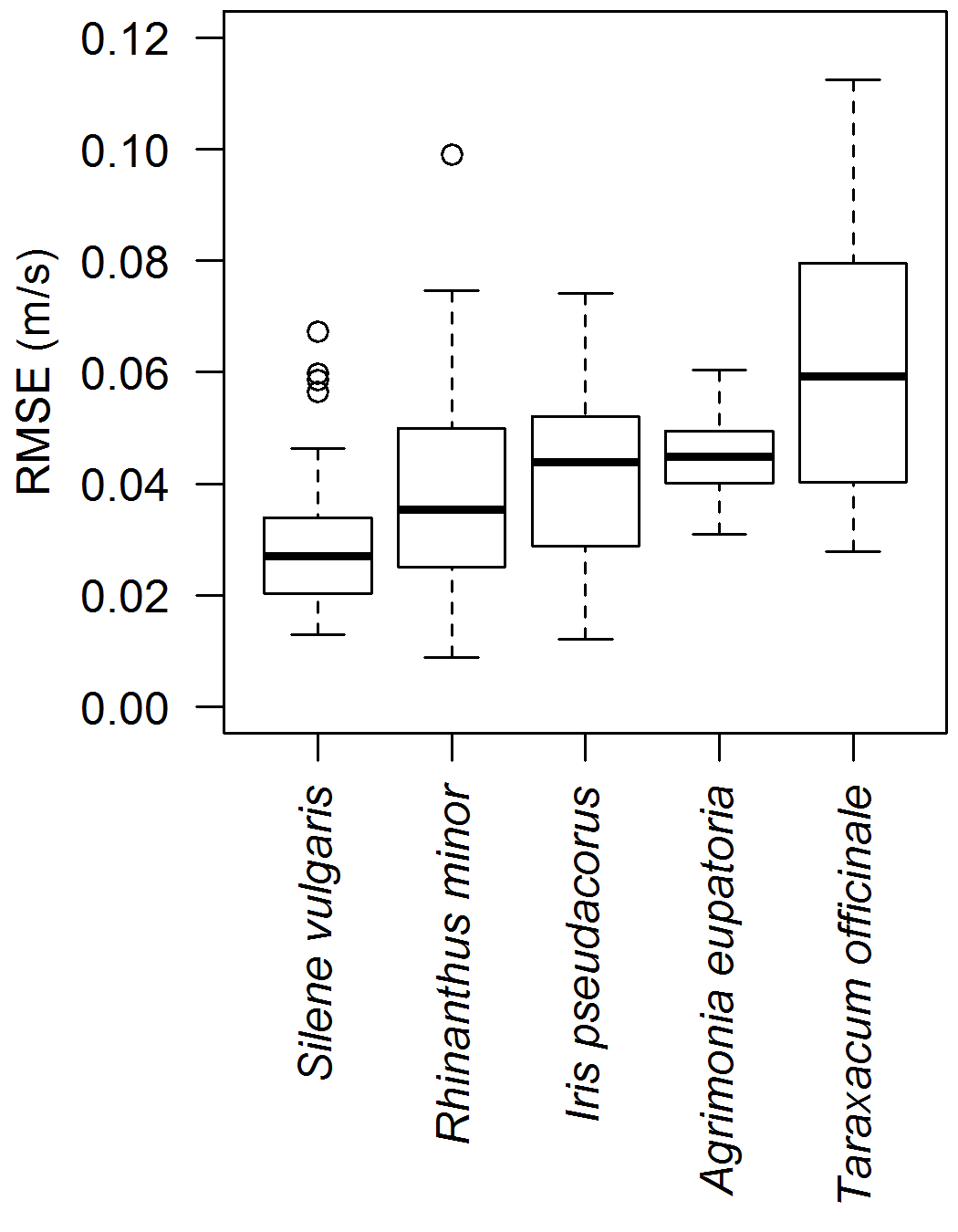


**Fig. S6**. Root-mean-square error (RMSE) of differences between observed and predicted velocity between successive images for each study species. Predicted velocities were derived from the physical model for free fall with acceleration fitted to the vertical seed trajectory.


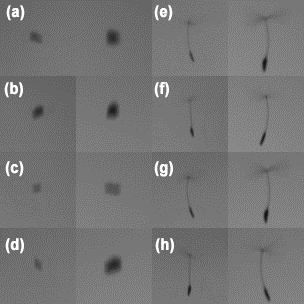


**Fig. S7**. Variation in the falling behaviour of the same seed between four replicate seed releases in (a, b, c, d) *Rhinanthus minor* and (e, f, g, h) *Taraxacum officinale*. In each panel, the photo on the left shows the mirror view, and the photo on the right shows the front view. These photos were cropped from the videos.

**References**

Askew, A. P., Corker, D., Hodkinson, D. J., & Thompson, K. (1997). A new apparatus to measure the rate of fall of seeds. *Functional Ecology*, *11*(1), 121–125. doi:10.1046/j.1365-2435.1997.00049.x

Cheng, N. S. (2009). Comparison of formulas for drag coefficient and settling velocity of spherical particles. *Powder Technology*, *189*(3), 395–398. doi:10.1016/j.powtec.2008.07.006

Kattge, J., Bönisch, G., Díaz, S., Lavorel, S., Prentice, I. C., Leadley, P., … Wirth, C. (2020). TRY plant trait database – enhanced coverage and open access. *Global Change Biology*, *26*(1), 119–188. doi:10.1111/gcb.14904

Nathan, R., Katul, G. G., Bohrer, G., Kuparinen, A., Soons, M. B., Thompson, S. E., … Horn, H. S. (2011). Mechanistic models of seed dispersal by wind. *Theoretical Ecology*, *4*(2), 113–132. doi:10.1007/s12080-011-0115-3

Taylor, J. R. (2005). *Classical mechanics*.

Vogel, S. (1994). *Life in Moving Fluids*. Princeton University Press.
